# Reduced Reward Responsiveness in Women With Moderate - to - Severe Premenstrual Syndrome: Evidence From a Probabilistic Reward Task

**DOI:** 10.3389/fpsyt.2020.00028

**Published:** 2020-02-13

**Authors:** Lulu Hou, Lei Chang, Lirong Chen, Renlai Zhou

**Affiliations:** ^1^Department of Psychology, Nanjing University, Nanjing, China; ^2^Department of Psychology, Faculty of Social Sciences, University of Macau, Macau, China

**Keywords:** premenstrual syndrome, positive affect, reward processing, menstrual cycle, late luteal phase

## Abstract

Nearly 50% of women of reproductive age worldwide experience premenstrual syndrome (PMS). Women with PMS exhibit low positive affect and low frontal electroencephalography asymmetry scores, both of which are associated with reward processing. These findings suggest that women with PMS may exhibit deficiencies in reward processing. A probabilistic reward task based on signal detection approach was used to assess reward responsiveness in 30 women with moderate-to-severe PMS and 31 controls without PMS. The results revealed that in the late luteal phase, the women with moderate-to-severe PMS exhibited lower response bias and lower hit rate toward more frequently rewarded stimuli (rich stimuli) than the controls. By contrast, the response bias and hit rate did not differ between the two groups in the follicular phase. The group differences still remained after controlling for anhedonic symptoms. Furthermore, trial-by-trial probability analyses revealed that women with moderate-to-severe PMS exhibited a trend of having a higher miss rate for rich stimuli than the controls. In particular, when a rich stimulus was preceded by an infrequently rewarded stimulus (a rewarded lean stimulus), participants in the PMS group exhibited a trend for higher miss rate than those in the control group in the late luteal and follicular phases. However, group differences in the probability analyses were nonsignificant after controlling for anhedonic symptoms. These results provide preliminary evidence that women with moderate-to-severe PMS exhibit dysfunctional reward responsiveness and impaired ability to modulate their behavior as a function of prior reinforcement.

## Introduction

Premenstrual syndrome (PMS) refers to a set of physical, emotional, and behavioral symptoms that occur periodically in women during the late luteal phase of the menstrual cycle, peaking within the week preceding menses and improving or disappearing after the onset of menses (1–3). A meta-analysis study revealed the prevalence rate of PMS is as high as 47.8% (4). Given the high proportion of women who experience PMS, its etiology has attracted considerable attention among researchers in recent years (5–8).

Most studies have focused on the increase in negative emotions of women with PMS [e.g., (5, 6)]. However, several studies have revealed that some women with PMS experience abnormal emotional reactions to positive events in daily life and exhibit deficiencies in self-reported physiological response to positive stimuli under laboratory conditions during the late luteal phase. For example, in a study by Rubinow et al. (9), 20 women with PMS and eight women without PMS were asked to use a bipolar visual analogue scale to record their emotional states over two consecutive cycles. The results revealed a significant difference between the two groups 3 days before the onset of menses. Subsequently, Metcalf and Livesey (10) used a similar method to record the emotional changes in 48 women with PMS and 44 without PMS throughout their menstrual cycle. Cluster analysis revealed that in the women with PMS, positive affect peaked on the 11th day of the menstrual cycle, whereas the women without PMS did not exhibit any significant peak. Recently, Chen and Zhou (11) used a picture-viewing paradigm and observed that the intensity of positive responses to pleasant images gradually declined in a group of women with PMS 400 ms to 800 ms after picture presentation. By contrast, the intensity of positive responses to the same images in a group of women without PMS exhibited an increasing trend. Taken together, these findings provide evidence that PMS is characterized by low positive affect.

Over the years, substantial evidence suggesting that positive affect is closely related to rewards has accumulated. When people are rewarded, they commonly experience positive affect (12). The creation of positive affect is related to the activation of reward pathways in the brain. Listening to pleasant music (13, 14) or viewing representations of love (15) has been reported to activate reward circuits in the brain. In addition, the amygdala, the brain area associated with emotions, projects extensively to other brain areas, such as the ventral striatum (16) and ventral tegmental area (17), both of which play key roles in reward processing (18). Therefore, dysfunctional reward processing may be associated with low positive affect.

Furthermore, studies measuring resting brain electrical activity have reported that PMS is characterized by relatively low frontal electroencephalography (EEG) asymmetry scores (calculated as ln [right alpha] - ln [left alpha]), which have been proven to be associated with reward responsiveness (12, 19). Deng et al. (20) reported that women with PMS had lower frontal alpha asymmetry scores during the resting state than those without PMS. Liu et al. (21) reported that the frontal EEG asymmetry scores were positive among women without PMS and negative among women with PMS. In addition, the score in the PMS group shifted from negative to positive after biofeedback training. Sutton and Davidson (22) reported that participants with higher resting left-sided frontal activity (i.e., higher EEG asymmetry scores) selected more pleasant stimuli in a later judgment task than those with higher resting right-sided frontal activity (i.e., lower EEG asymmetry scores). Pizzagalli et al. (23) recorded the resting EEG of 18 participants who separately performed a verbal memory task under three incentive conditions (neutral, reward, and punishment). The results indicated that higher resting frontal EEG asymmetry scores were associated with a stronger bias to respond to reward-related cues.

Although the strong associations between PMS and lower positive affect (9) and between PMS and lower resting frontal asymmetry scores (21) are known and both factors are reportedly linked to a dysfunction in reward processing (12, 23), few studies have specifically assessed the association between PMS and reduced reward processing. Pizzagalli et al. (19) developed a probabilistic reward task (PRT) to study reward responsiveness. In the task, signal detection theory was used to measure the response bias to stimuli with different reward probabilities. The task involved two stimulus types, and one of the stimulus has three times higher likelihood of reward than the other. In addition, the task unveils two indicators, namely discrimination and response bias. Discrimination reflected the difficulty of the task, and response bias reflected the tendency of individuals to recognize one type of stimulus as the other. Individuals who identified a less frequently rewarded stimulus (lean stimulus) as a more frequently rewarded stimulus (rich stimulus) had a high reward response bias, whereas those who identified a rich stimulus as a lean stimulus had a low response bias. This paradigm reflects an individual's response bias to reward stimuli and has been used extensively among healthy populations (19, 24, 25), patients with major depressive disorders (MDD), and patients with bipolar disorder [BPD; (26, 27)]. For example, Pizzagalli et al. (26) demonstrated that patients with MDD exhibited lower reward responsiveness than healthy controls. Another study also showed that the euthymic BPD group exhibited a lower and more delayed acquisition of response bias toward a rich stimulus than healthy controls (27).

On the basis of the aforementioned findings, we used the PRT to examine reward responsiveness in women with moderate-to-severe PMS. Considering the characteristics of low positive affect and low frontal EEG asymmetry scores in women with PMS, we hypothesized that women with moderate-to-severe PMS would exhibit deficiencies in reward processing and that consequently, their response bias to a rich stimulus would be lower than that of women without PMS during the late luteal phase. Furthermore, with reference to the analytical methods adopted in other studies (26, 27), we conducted a probabilistic analysis. Specifically, blunted reward responsiveness emerges if participants have (1) low rates of correct identification (hits) for the rich stimulus, and/or (2) low rates of incorrect identification (misses) for the lean stimulus. If (1) was observed, then we computed the probability of missing a rich stimulus as a function of the outcome in the immediately preceding trial. If (2) was observed, then we computed the probability of missing a lean stimulus as a function of the outcome in the immediately preceding trial. By conducting probabilistic analysis, Pizzagalli et al. (26) discovered that in an MDD sample, blunted response bias was mainly caused by a low miss rate for lean stimuli when they were immediately preceded by a rich stimulus with no reward feedback. However, Pizzagalli et al. (27) found that reduced response bias in BPD patients was due to a low hit rate for rich stimuli when they were immediately preceded by a rewarded lean stimulus. In summary, patients with MDD and those with BPD exhibit reduced reward response bias, but the reasons underlying their response are different. For one, the reason is that reward reinforcement cannot be maintained in the absence of an immediate reward, and for the other, the reason is that reward reinforcement is replaced because of increased sensitivity to single rewards of the disadvantageous stimulus. Therefore, the probabilistic analysis plays a crucial role in our understanding of the specific reason for the reduced reward responsiveness in women with PMS. Finally, to determine if altered reward responsiveness represents a stable vulnerability to PMS that is not caused by differences in other variables (particularly anhedonia), we also evaluated whether the group differences remained even when statistically controlling for Snaith–Hamilton Pleasure Scale (SHAPS) scores, which are used to assess altered reward processing (26).

## Materials and Methods

### Participants

In accordance with Bancroft's recommendations (1), a PMS scale that had been translated into Chinese (28) was used in the present study for sample selection alongside posters at Nanjing University and online advertisements. The participants were given 50 yuan after completing the task twice. Of the 331 women who completed the PMS scale, the prevalence of mild, moderate, and severe PMS was 33.8% (*n* = 112), 15.7% (*n* = 52), and 2.1% (*n* = 7), respectively. Therefore, 59 female college students met the diagnostic criteria for moderate-to-severe PMS. Of the 59, 19 participants were excluded from the study because of invalid contact information (*n* = 3), because they were not willing to participate in the experiment after learning the details (*n* = 4), because they did not have enough free time to participate twice (*n* = 7, two of them attended only once, and the other five did not attend at all), or because the length of their menstrual cycle was either not fixed or too long (*n* = 5). Forty women with moderate-to-severe PMS and 40 without PMS volunteered to participate in our experiment after matching BMI and age.

All enrolled participants were undergraduate or postgraduate students, had a fixed menstrual cycle (25–35 days without fluctuation of more than 3 days in the preceding 6 months), had no reproductive history, did not use contraceptives, had no self-reported personal history of diagnosed psychiatric disorders, and presented no severe anxiety and depression tendency as determined by Beck Depression Inventory (BDI) and Beck Anxiety Inventory (BAI) scores. Furthermore, of the 80 participants, 19 were excluded because of an excessive number of outliers (*n* = 14), response repetition (*n* = 2), or a misunderstanding of task instructions (*n* = 3). Specifically, outliers refer to trials with RTs less than 150 ms or longer than 1500 ms and those with RTs (following natural log transformation) falling outside the mean ±3 standard deviation. Participants whose number of outliers per block exceeded 20 or whose overall number of outliers exceeded 60 were removed. Overall, 4.98% of all trials in this study were excluded. Response repetition means pressing the same button at least 15 times consecutively and is suggestive of a participant having not taken the task seriously. Misunderstanding the task instructions means that the accuracy of at least one block was lower than 55% (i.e., chance performance). Finally, data from 30 women with moderate-to-severe PMS (PMS scale scores: 14.77 ± 3.87) and 31 women without PMS (PMS scale scores: 3.19 ± 1.44) were used for analysis. The PMS scale scores of the women with moderate-to-severe PMS were higher than those of the women without PMS [*t* (59) = 15.58, *p* < 0.001]. Notably, two women in the PMS group met the diagnostic criteria of premenstrual dysphoric disorder (PMDD) based on the premenstrual symptoms screening tool (PSST). Therefore, we have provided the results excluding these two participants in the **Supplementary Materials** although the difference was minor.

### Materials

#### PMS Scale

The Chinese version (28) of the PMS scale (1) was used to measure individuals' PMS severity. The scale consists of 12 items covering physical and psychological symptoms (e.g., depression, anxiety, inattention) and is used to investigate PMS symptoms in women 14 days before their most recent episode of menstruation. The participants rated statements on a 4-point scale from 0–3 (0 = no symptoms; 1 = mild symptoms; 2 = the symptom has some impact on everyday work and life, but can be endured; 3 = the symptom severely affects daily life, study, and work, and thus requires treatment) to indicate the extent to which each item was representative of their symptoms. A score of 6–10 indicated mild PMS, 11–20 indicated moderate PMS, and more than 20 indicated severe PMS. The Cronbach's alpha = 0.80 in Wu et al. (29) and 0.91 in the present study. Furthermore, the scale was deemed valid in differentiating women with PMS from those without PMS (21, 29–31) and reflected PMS severity (32, 33).

#### Premenstrual Symptoms Screening Tool

The Chinese version (34) of the PSST (35) was used to identify women experiencing PMDD. The scale operationalizes DSM-IV criteria for PMDD and consists of 19 items. The first 14 items measure the severity of symptoms, and the last 5 measure the influence of symptoms. The participants rated statements on a 4-point scale from 0 (not at all) to 3 (severe) to indicate the extent to which each item was representative of their symptoms. The following criteria had to be met for PMDD to be diagnosed: 1) at least one of the first 4 items is rated as severe; 2) in addition, at least four of the first 14 items are rated as moderate to severe; and 3) at least one of the last 5 items is rated as severe. The Cronbach's alpha = 0.92 in Hou et al. (34) and 0.92 in the present study.

#### Beck Depression Inventory

The Chinese version (36) of the BDI (37) was used to measure individuals’ level of depression. The scale consists of 21 items. The participants rated statements on a 4-point scale from 0 (no) to 3 (extremely severe) to indicate the extent to which each item was representative of their symptoms. The Cronbach’s alpha = 0.85 and test–retest reliability = 0.73 after 1 week. A score ≤4 indicated no depression, 5–13 indicated mild depression, 14–20 indicated moderate depression, and ≥21 indicated severe depression. In addition, the scale had Cronbach's alpha values of 0.87 and 0.92 in the late luteal phase and follicular phase, respectively, in the present study.

#### Beck Anxiety Inventory

The Chinese version (38) of the BAI (39) was used to measure individuals' level of anxiety. The scale consists of 21 items. The participants rated statements on a 4-point scale from 1 (no) to 4 (extremely severe) to indicate the extent to which each item was representative of their symptoms. The BAI scores were standard scores, which were obtained by Y = int (1.19X), where X represents the raw scores. A score ≥45 indicated severe anxiety. The Cronbach's alpha = 0.95 in Zheng et al. (38) and 0.89 and 0.91 in the late luteal phase and follicular phase, respectively, in the present study.

#### Positive and Negative Affect Schedule

The Chinese version (40) of the Positive and Negative Affect Schedule [PANAS; (41)] was used to measure the participants’ affective state. The scale consists of 20 items and two subdimensions for positive affect (PA subdimension) and negative affect (NA subdimension). The participants rated statements on a 5-point scale from 1 (not at all) to 5 (very much) to indicate the extent to which each statement applied to them. The reliability of the scale was as follows: Cronbach's alpha of PA and NA subdimensions = 0. 83 and 0. 85, respectively; test–retest reliability of PA and NA subdimensions = 0.47 and 0.47, respectively, after 4 weeks. In the present study, the PA subdimension had Cronbach's alpha scores of 0.92 and 0.89 in the late luteal phase and follicular phase, respectively. The NA subdimension had Cronbach's alpha scores of 0.87 and 0.94 in the late luteal phase and follicular phase, respectively.

#### Brief Profile of Mood States

The Chinese version (42) of the Brief Profile of Mood States [BPOMS; (43)] was used to measure the participants' mood states. We used the vigor–activity (VA) subdimension to measure the participants’ mood states. The BPOMS-VA consists of five items. The participants rated statements on a 5-point scale from 0 (not at all) to 4 (very much) to indicate the extent to which each statement applied to them. The reliability of the scale was as follows: Cronbach’s alpha = 0.88; test-retest reliability = 0.62 after 9 days, and the scale had Cronbach's alpha scores of 0.91 and 0.88 in the late luteal phase and follicular phase, respectively.

#### Basic Emotion Experience Scale

Following Wu et al. (44), the Basic Emotion Experience Scale (BEES) was used to measure the participants’ basic emotions. Of the nine items on the scale, two were used to measure general valence and arousal and the other seven were used to measure seven basic emotions (joy, anger, fear, sadness, calm, disgust, and surprise). The participants rated statements on a 5-point scale from 0 (very strongly disagree) to 9 (very strongly agree) to indicate the extent to which each statement applied to them.

#### Snaith–Hamilton Pleasure Scale

The Chinese version (45) of the Snaith–Hamilton Pleasure Scale [SHAPS; (46)] was used to measure four domains of hedonic experience, namely interests and pastimes, social interaction, sensory experience, and diet. The scale consists of 14 items. The participants rated statements on a 4-point scale from 1 (completely agree) to 4 (completely disagree) to indicate the extent to which each item represented their experiences. The reliability of the scale was as follows: Cronbach's alpha = 0.85 and test–retest reliability = 0.64 after 4 weeks in Liu et al. (45), and Cronbach's alpha scores = 0.86 and 0.82 in the late luteal phase and follicular phase, respectively, in the present study.

#### PRT

**Figure 1:**
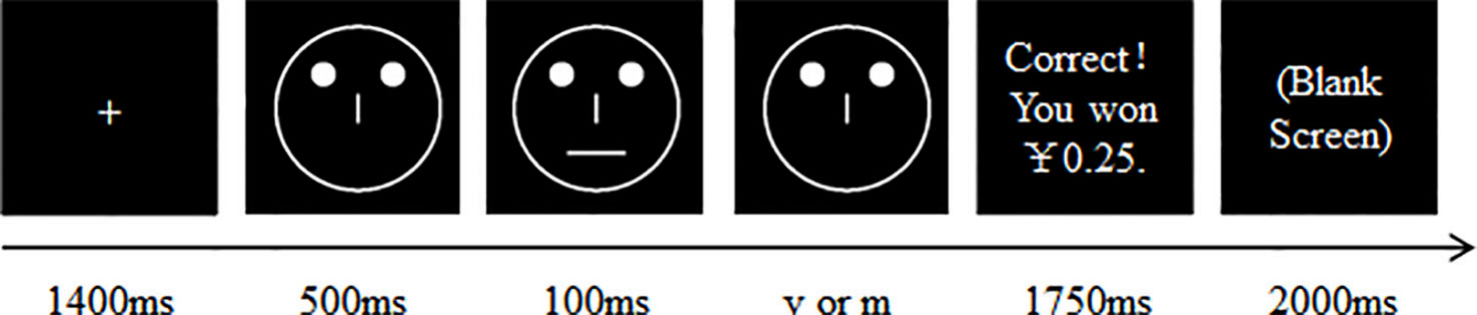
The time course for a single trial. For each trial, the participant was asked to decide whether a short or a long mouth (or nose) was presented by pressing either the ‘v’ or the ‘m’ key of a PC keyboard. The version of the PRT used, the rich stimulus type, and the keyboard keys mapped to the rich and lean stimuli were counterbalanced among the participants.

As illustrated in **Figure 1**, for the PRT, each trial began with the presentation of a fixation cross in the center of the screen for 1400 ms. The cross was then replaced by a mouthless (or noseless) face displayed in the center of the screen for 500 ms. Subsequently, a face with a short mouth (10.00 mm) or nose (5.00 mm) or long mouth (11.00 mm) or nose (5.31 mm) was presented for 100 ms. After the mouth (or nose) had disappeared, the mouthless (or noseless) face remained on the screen for an additional 1500 ms. The participants were instructed to identify which stimulus (long or short) was presented by pressing the ‘v’ or ‘m’ key on the keyboard, respectively. In all the trials, some of the correct identifications were followed by a reward feedback (“Correct!! You have won 0.25 Yuan”), which was presented for 1500 ms followed by a blank screen for 2000 ms. If no feedback was given (i.e., the participant’s response was inaccurate or was accurate, but no feedback was scheduled), a blank screen was displayed for 3500 ms.

The task consisted of three blocks comprising 100 trials. In each block, short and long stimuli were presented equally in a pseudo-randomized sequence. Notably, no more than three instances of the same stimulus were presented consecutively. Additionally, in each block, reward feedback was given after 40 correct trials according to a controlled reinforcement schedule in a pseudo-randomized sequence. Critically, one stimulus type (i.e., rich stimulus) was rewarded three times more frequently than the other (i.e., lean stimulus). If a participant failed to give a correct response in a trial where feedback was scheduled, reward feedback was delayed until the next correct identification of the same stimulus type.

Notably, before the experiment began, the participants were informed that their performance on the task would be accounted for in their final income. They were thus required to make judgments as quickly and accurately as possible to earn as much money as possible. In addition, before the experiment, the participants were informed that not all correct identifications would be rewarded. However, they were not told that one type of stimulus would be rewarded more frequently than the other. Crucially, because the participants were required to complete the task twice (in the late luteal phase and follicular phase), the version of the PRT used (nose or mouth), the rich stimulus type (long or short), and the keyboard keys mapped to the rich and lean stimuli (“v” and “m,” respectively) were counterbalanced among the participants. Importantly, the differences of task difficulty (e.g. discriminability) between the nose and mouth versions of PRT were minor through the pilot study (24). Furthermore, the total amount of money income received by the participants is a certain amount, and the income received twice is slightly different (have been counterbalanced among participants). Because the probability of the participants receiving the rewards is 40% (30 for rich stimuli and 10 for lean stimuli for each block) and their accuracy is much higher than 40%, generally speaking, the amount of money income they receive is basically the same. We didn't give an explanation right after the experiment because they didn't complete the experiment at the same time (the time of each person's menstruation is different). If we give an immediate explanation, it is likely to disclose the purpose of the experiment and influence the results of the experiment.

### Procedure

From the time of menstrual onset and length of menstrual cycle reported by the participants in the early screening process, the time of menstrual onset was deduced. Each participant was required to participate twice: once in the late luteal phase (1–4 days before menses onset) and once in the follicular phase (1–4 days after menses onset). The phases during which the participants participated in the experiment for the first time were counterbalanced among the participants. After arriving at the laboratory, each participant first signed an informed consent form (only at the first visit) and then completed the questionnaires (BDI, BAI, PANAS, BPOMS-VA, BEES, and SHAPS), and finally performed the PRT. Saliva was collected from all participants before they left the laboratory.

### Biochemical Assays

The participants were asked to avoid foods high in fat and protein as well as alcohol the day before sampling. In addition, no food or water was to be consumed within 30 minutes before sampling. All saliva samples were collected using a Cayman sampling device and stored at –20°C until assay. Estradiol and progesterone analyses were conducted using competitive enzyme-linked immunosorbent assay. All intra- and inter-assay coefficients of variation were below 12%.

### Data Analysis

#### Demographic Variables and Scales

First, we used independent-sample *t* tests to evaluate the differences between the demographic variables of the two groups. Second, for each self-reported measure of affect (PMS scale, PANAS-PA, BPOMS-VA, BEES, and SHAPS), an analysis of variance (ANOVA) with Phase (late luteal phase, follicular phase) as repeated measures and Group (PMS group, non-PMS group) as a between-subject factor was performed. Where necessary, Greenhouse–Geisser correction was used. Effect sizes are reported as *η_p_^2^* and *Cohen’s d* values.

#### PRT

First, task performance was assessed by computing response bias, discriminability, hit rates, and reaction times. Response bias and discriminability were calculated using the following formulae:

$$\text{Response bias}:\;\log b=\frac{1}{2}log\left(\frac{Rich_{correct}\times Lean_{incorrect}}{Rich_{incorrect}\times Lean_{correct}}\right)$$

$$\text{Discriminability}:\log d=\frac{1}{2}log\left(\frac{Rich_{correct}\times Lean_{correct}}{Rich_{incorrect}\times Lean_{incorrect}}\right)$$

Subsequently, to measure response bias and discriminability, a separate ANOVA with Phase (late luteal phase, follicular phase) and Block (Block 1, Block 2, Block 3) ^1^ as repeated measures and Group (PMS group, non-PMS group) as a between-subject factor was performed. Furthermore, to measure the hit rates and reaction times, another ANOVA with Phase (late luteal phase, follicular phase), Stimulus type (rich, lean), and Block (Block 1, Block 2, Block 3) as repeated measures and Group (PMS group, non-PMS group) as a between-subject factor was performed. Finally, following methods used in previous studies (26, 27), probability analyses were performed. Specifically, we computed the probability of missing a rich or lean stimulus as a function of the outcome of the preceding trial. Specifically, to calculate the rich stimulus or lean stimulus missing rates, an ANOVA with Phase (late luteal phase, follicular phase) and Preceding trial type (reward rich, non-reward rich, reward lean, non-reward lean) as repeated measures and Group (PMS group, non-PMS group) as a between-subject factor was performed. Before statistical analyses were conducted, probability values were arcsine transformed. Moreover, to test whether group differences remain significant when accounting for anhedonia (as measured by SHAPS), all results involving group differences were further analyzed by using analysis of covariance (ANCOVA) with the SHAPS scores in the late luteal phase and follicular phase, respectively, as covariates. Where necessary, Greenhouse–Geisser correction was used. Significant findings underwent follow-up assessment with the Bonferroni *post hoc* test. Effect sizes are reported as *η_p_^2^* and *Cohen’s d* values.

## Results

### Demographic Variables

The results revealed no group differences for age, BMI, duration of menstrual flow, or length of menstrual cycle [all *t*(59) < 1.55, *p* > 0.05]. The descriptive statistics of demographic variables are listed in **Table 1**.

**Table 1:** Demographic variables and PMS scale in two group (M ± SD).

	PMS group (*n* = 30)	Non-PMS group (*n* = 31)	*t*
Age	21.03 ± 2.11	21.87 ± 2.14	-1.54
BMI	19.88 ± 1.36	20.08 ± 2.01	-0.45
Duration of menstrual flow (days)	5.67 ± 1.09	5.39 ± 1.05	1.02
Length of menstrual cycle (days)	29.80 ± 2.37	29.16 ± 3.14	0.89

BMI refers to body mass index, which was calculated as the participant's weight in kilograms divided by the square of her height in meters (kg/m^2^). Duration of menstrual flow here refers to the duration of the menstrual phase in a single menstrual cycle. Length of menstrual cycle here refers to the interval between two consecutive menstrual cycles.

### Questionnaires and Hormones

No significant effects were observed for PANAS-PA [all *F*(1,59) < 2.68, *p* > 0.20] or BPOMS-VA [all *F*(1,59) < 2.29, *p* > 0.14] or BEES–general arousal [all *F*(1, 59) < 1.60, *p* > 0.21]. The ANOVA for BEES–general valence revealed a main effect for Group [*F* (1, 59) = 13.00, *p* = 0.001, *η_p_^2^* = 0.18]. The ANOVA for the SHAPS revealed a main effect for Group [*F*(1, 59) = 7.48, *p* = 0.008, *η_p_^2^* = 0.11]. The scores of women with moderate-to-severe PMS were higher than those of women without PMS.

The hormone level results revealed significant main effects of Phase on progesterone [*F*(1, 59) = 42.24, *p* < 0.001, *η_p_^2^* = 0.42] and estradiol [*F*(1, 59) = 6.68, *p* = 0.01, *η_p_^2^* = 0.10]. However, no other effects were observed for these hormones [progesterone: all *F*(1,59) < 0.01, *p* > 0.96; estradiol: all *F*(1,59) < 0.71, *p* > 0.40]. The questionnaires and hormones in the two groups are presented in **Table 2**.

**Table 2:** Questionnaires and hormones in two group (M ± SD).

	PMS group (*n* = 30)		Non-PMS group (*n* = 31)	
	late luteal phase	follicular phase	late luteal phase	follicular phase
BDI	15.43 ± 7.18	15.53 ± 10.18	7.42 ± 6.38	7.07 ± 5.81
BAI	44.20 ± 10.73	43.80 ± 11.20	33.39 ± 4.61	32.97 ± 5.78
PANAS-N	25.83 ± 6.01	24.53 ± 5.45	20.87 ± 3.51	21.52 ± 4.63
PANAS-P	23.63 ± 4.89	22.80 ± 4.65	21.74 ± 4.11	22.55 ± 4.85
BPOMS-VA	6.47 ± 3.29	7.23 ± 3.85	6.37 ± 4.57	8.03 ± 4.18
BEES-general valence	4.50 ± 1.70	4.93 ± 1.62	5.68 ± 1.49	5.94 ± 1.12
BEES-general arousal	4.93 ± 1.38	4.80 ± 1.49	4.94 ± 0.93	5.32 ± 1.05
BEES-joy	4.77 ± 1.76	4.57 ± 1.74	5.81 ± 1.35	5.81 ± 1.62
BEES-anger	4.70 ± 1.60	5.03 ± 1.87	3.10 ± 1.14	3.19 ± 1.38
BEES-fear	4.17 ± 1.66	4.60 ± 2.14	3.29 ± 1.44	2.94 ± 1.57
BEES-sadness	5.10 ± 1.75	5.00 ± 1.86	3.87 ± 1.65	3.90 ± 1.89
BEES-calm	4.43 ± 1.85	4.27 ± 1.66	5.58 ± 1.26	5.55 ± 1.79
BEES-disgust	5.00 ± 1.50	4.60 ± 1.99	3.13 ± 1.41	3.48 ± 1.77
BEES-surprise	4.73 ± 1.78	4.27 ± 1.55	4.03 ± 1.74	4.29 ± 1.62
SHAPS	25.27 ± 5.21	25.73 ± 4.52	22.00 ± 4.56	22.81 ± 5.11
progesterone (pg/mL)	841.49 ± 593.48	373.48 ± 177.43	967.75 ± 587.83	492.79 ± 306.02
estradiol (pg/mL)	130.76 ± 102.12	100.58 ± 70.85	146.36 ± 83.74	114.56 ± 71.56

### PRT

### Response Bias

**Figure 2:**
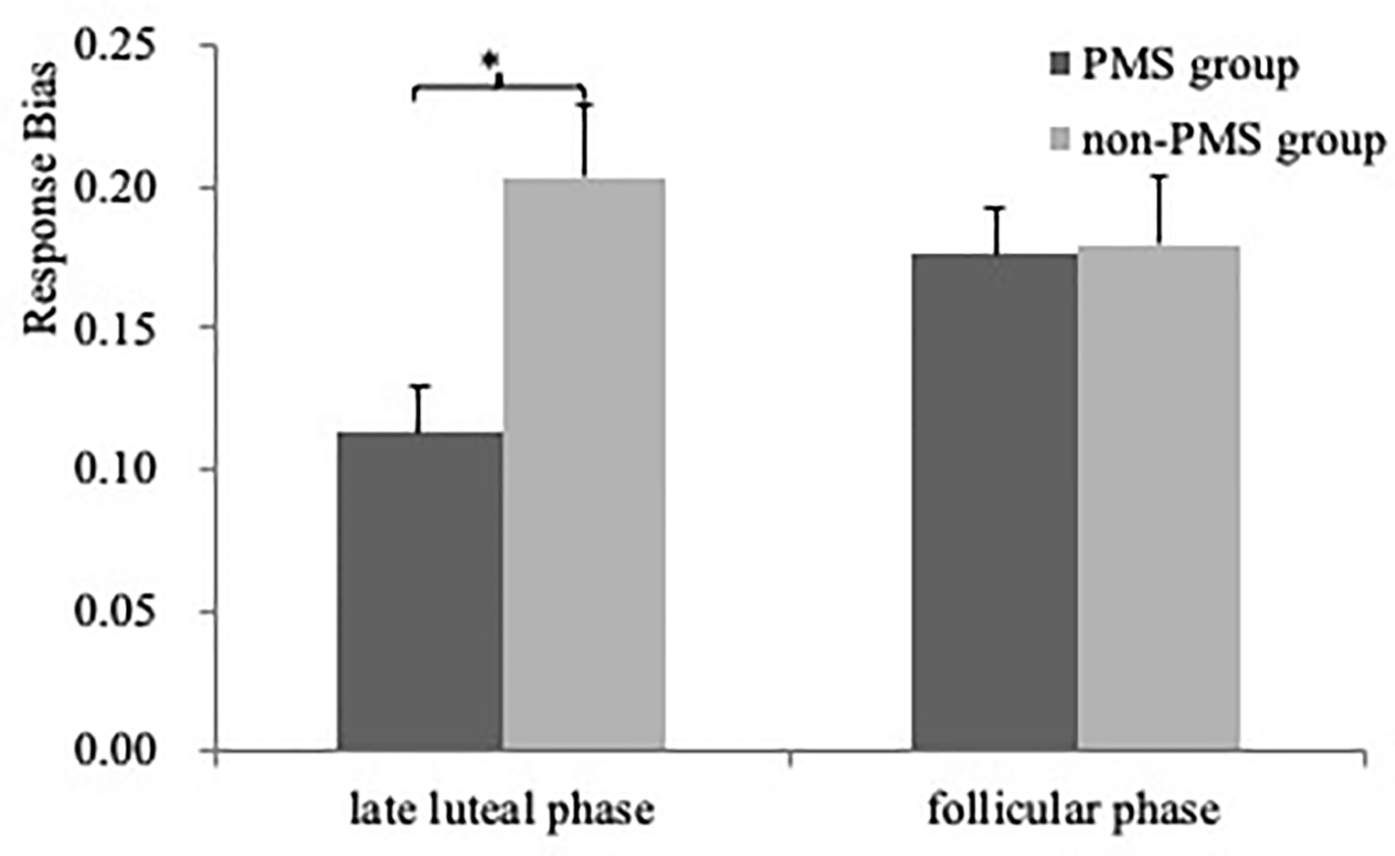
Response bias (averaged across the three blocks) as a function of Phase (late luteal phase and follicular phase) for PMS group (n = 30) and non-PMS group (n = 31). Error bars represent standard errors. *represents p < 0.05.

As illustrated in **Figure 2**, One ANOVA revealed a significant effect for Block [*F*(2, 118) = 43.94, *P* < 0.001, $H_{P}^{2}=0.43$] resulting from significantly higher response bias in Blocks 2 and 3 than in Block 1 (Bonferroni *P* < 0.05). The main effect for Group [*F*(1, 59) = 5.94, *P* = 0.02, $H_{P}^{2}=0.09$] was also significant owing to higher response bias in the women without PMS than in those with moderate-to-severe PMS (0.19 ± 0.06 Vs. 0.15 ± 0.09; *Cohen’s D* = 0.52). Furthermore, although the same ANOVA only revealed a trend of Group × Phase interaction [*F*(1, 59) = 3.58, *P* = 0.06], in the late luteal phase, the women without PMS had a higher response bias than those with moderate-to-severe PMS [0.20 ± 0.08 Vs. 0.11 ± 0.10; *F*(1, 59) = 15.11, *P* < 0.001, *Cohen’s D* = 0.99], whereas in the follicular phase, no difference was observed between the two groups [*F*(1, 59) = 0.01, *P* = 0.93]. No other significant effects were noted (all *F* < 0.89, *P* > 0.40).

The results of the ANCOVA for the response bias in the late luteal phase indicated that the main effect for Group [*F*(1, 58) = 11.14, *p* = 0.001, *η_p_^2^* = 0.16] was significant owing to higher response bias in the women without PMS than in those with moderate-to-severe PMS. No other significant effects were noted (all *F* < 1.55, *p* > 0.22). For the response bias in the follicular phase, no significant effects were observed (all *F* < 3.74, *p* > 0.06).

#### Discriminability

No effects involving Group emerged (all *F* < 0.88, *p* > 0.35).

#### Reaction Time

One ANOVA revealed a significant Group × Phase × Block three-way interaction [*F*(1, 59) = 4.40, *p* = 0.01, *η_p_^2^* = 0.07]. However, simple effect tests revealed no further significant differences between the two groups for any condition (all *F* < 1.76, *p* > 0.19).

#### Hit Rate

One ANOVA revealed a significant main effect for Block [*F*(2, 118) = 5.27, *p* = 0.01, *η_p_^2^* = 0.08) driven by a significantly higher hit rate in Block 3 than in Block 1 (Bonferroni *p* < 0.05). Furthermore, the main effect for Stimulus Type was also significant [*F*(1, 59) = 263.68, *p* < 0.001, *η_p_^2^* = 0.82, rich stimulus > lean stimulus].

**Figure 3:**
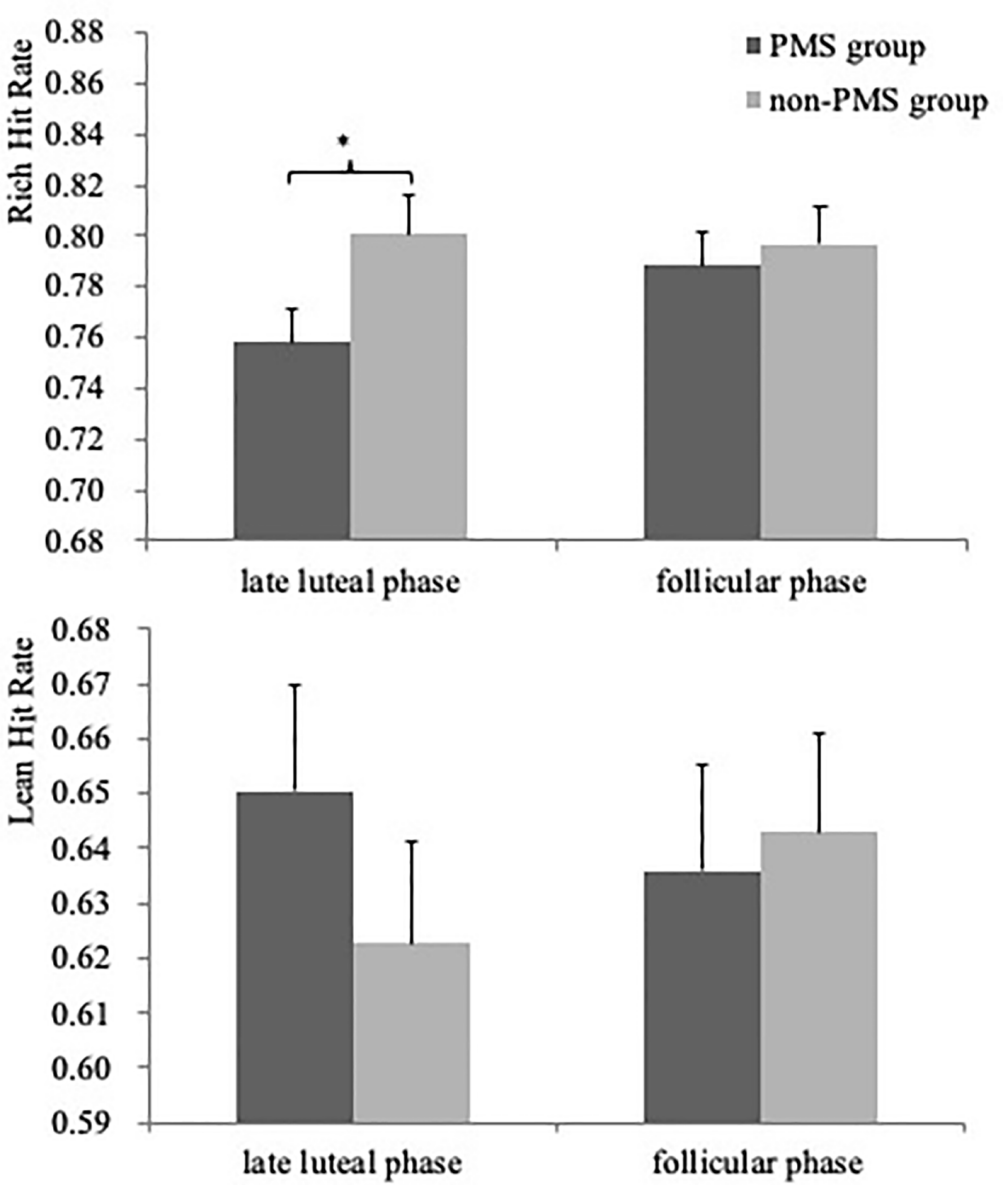
Mean accuracy (averaged across the three blocks) for the rich (top) and lean stimulus (bottom) as a function of Phase (late luteal phase and follicular phase) for PMS group (n = 30) and non-PMS group (n = 31). Error bars represent standard errors. *represents p < 0.05.

Critically, although the Group × Phase × Stimulus type interaction only revealed a trend [*F*(1, 59) = 3.06, *p* = 0.10], women without PMS had a higher hit rate to the rich stimulus in the late luteal phase than did women with moderate-to-severe PMS [0.80 ± 0.06 vs. 0.76 ± 0.08, *F*(1, 59) = 5.55, *p* = 0.02, *Cohen’s d* = 0.57], as illustrated in **Figure 3**. Moreover, a significant Group × Stimulus type interaction was observed [*F*(1, 59) = 3.97, *p* = 0.05, *η_p_^2^* ^=^ 0.06), but no group difference was noted in both Stimulus types (all *F* < 2.71, *p* > 0.11). No other significant effects were observed (all *F* < 0.89, *p* > 0.41).

The results of the ANCOVA for the hit rate in the late luteal phase indicated that the main effect of Stimulus type was significant [*F*(1, 58) = 11.61, *p* = 0.001, *η_p_^2^* = 0.17] owing to the higher hit rate of rich stimulus compared with that of lean stimulus. Furthermore, a significant Group × Stimulus type interaction was observed [*F*(1, 59) = 7.17, *p* = 0.01, *η_p_^2^* = 0.11]. The group difference was due to the rich stimulus. Specifically, women without PMS had a higher hit rate than did women with moderate-to-severe PMS [0.76 ± 0.08 vs. 0.80 ± 0.06, *F*(1, 59) = 5.55, *p* = 0.02, *Cohen's d* = 0.57]. No other significant effects were noted (all *F* < 1.28, *p* > 0.28). For the hit rate in the follicular phase, no significant effects were observed (all *F* < 2.67, *p* > 0.07).

#### Probability Analyses

The previous analyses indicated that women with moderate-to-severe PMS had significantly lower response bias and a significantly higher miss rate (i.e., lower hit rate) for the rich stimuli. To analyze these findings in more detail, we computed the probability of missing a rich stimulus as a function of the outcome of the preceding trial.

**Figure 4:**
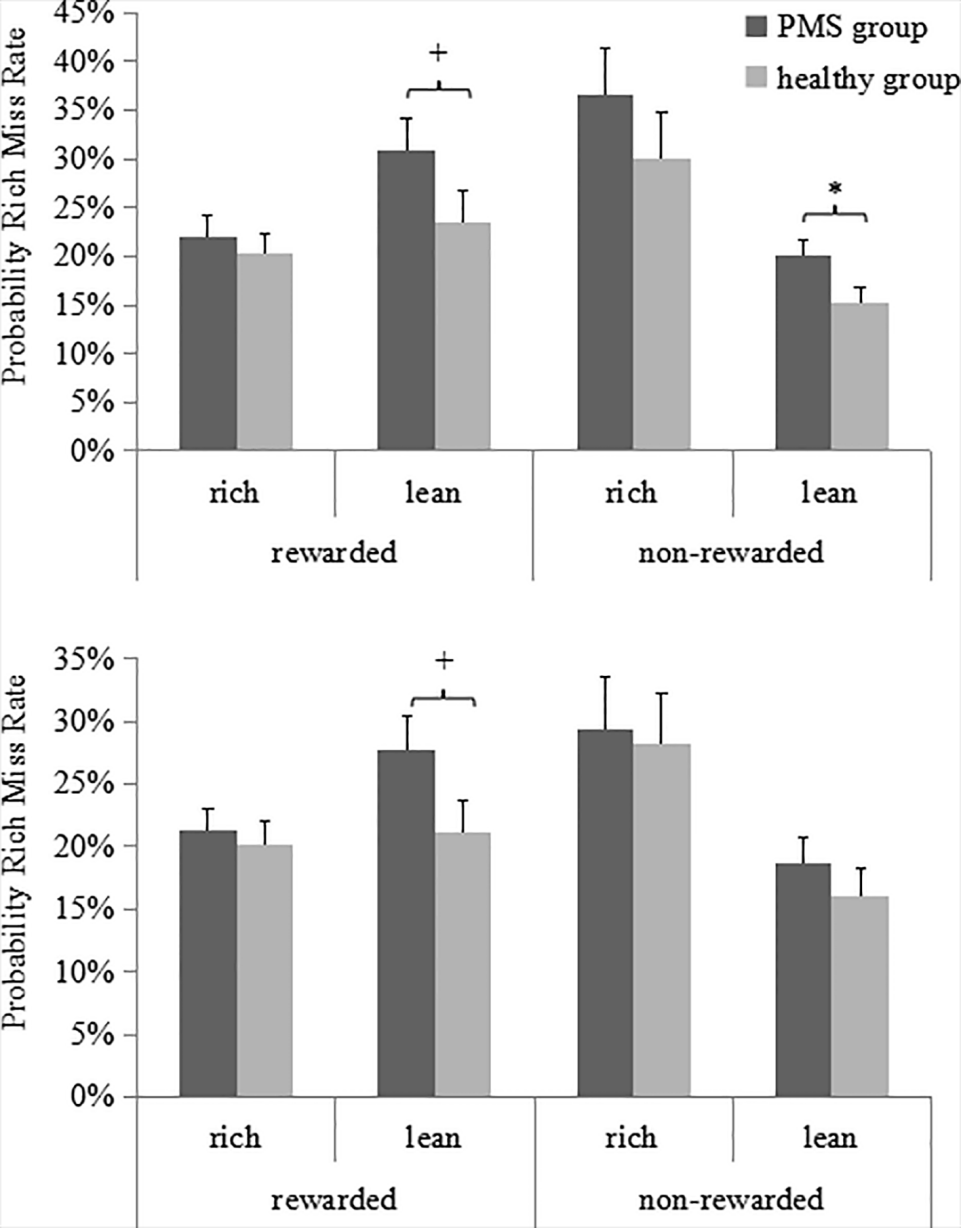
Probability of miss rates (averaged across the three blocks) for PMS group (n = 30) and non-PMS group (n = 31) as a function of whether the preceding rich or lean trial was rewarded or not in the late luteal (top) and follicular (bottom) phase. Error bars represent standard errors. *represents p < 0.05, ^+^represents p < 0.10.

As illustrated in **Figure 4**, the ANOVA for the rich stimulus miss rate revealed a significant main effect for Preceding trial type [*F*(1, 59) = 10.35, *p* = 0.002, *η_p_^2^* = 0.15]. Although the three-way interaction observed was nonsignificant, separate analyses indicated that in the late luteal phase, when the rich trial was preceded by a non-rewarded lean trial, the miss rate of the PMS group was higher than that of the non-PMS group [0.20 ± 0.06 vs. 0.15 ± 0.10; *t*(59) = 2.17, *p* = 0.03, *Cohen's d* = 0.61]. Furthermore, in both the late luteal and follicular phases, when the rich trial was preceded by a rewarded lean trial, the miss rate of the PMS group was higher than that of the non-PMS group [late luteal phase: 0.30 ± 0.17 vs. 0.23 ± 0.16, *t*(59) = 1.65, *p* = 0.10, *Cohen’s d* = 0.42; follicular phase: 0.27 ± 0.15 vs. 0.21 ± 0.12, *t*(59) = 1.85, *p* = 0.07, *Cohen's d* = 0.44]. No other significant effects were observed (all *F* < 1.25, *p* > 0.27). The results of the ANCOVA for all the probability analyses indicated no significant effects (all *F* < 1.78, *p* > 0.15).

## Discussion

This study explored the relationship between PMS and altered reward processing by asking women with moderate-to-severe PMS and without PMS to complete a PRT in the late luteal and follicular phases. The results indicated that moderate-to-severe PMS is characterized by an impaired ability to modulate behavior as a function of prior reinforcement history. Reinforcement sensitivity theory characterizes three systems and is assumed to correspond to a series of neural pathways that underlie individual differences in personality and psychopathology (47). Among the three systems, the behavioral approach system motivates reward-seeking behaviors. When this system is activated, individuals experience positive affect. Because positive reinforcers are stimuli that increase the likelihood of specific behaviors (48), blunted responsiveness to positive reinforcers may reduce engagement in pleasurable activities as well as the motivation to seek rewards. In this study, the women with moderate-to-severe PMS were unable to modulate their behaviors according to differences in reward probabilities in the late luteal phase. Such dysfunction may result in the generation, maintenance, or exacerbation of symptoms (including emotional and behavioral symptoms) in the late luteal phase, thus leading to a lack of interest in one's environment and loss of pleasure. The results of this study are similar to those of previous studies on positive affect (9, 10) and frontal EEG asymmetry (20, 21) among women with PMS. Moreover, the present study provides evidence of the lack of motivation to seek reward stimuli among such women in the late luteal phase.

The main results of this study can be explained by two aspects. First, extensive evidence suggests that PMS is associated with increased daily stress and is exacerbated by stressful life events (49, 50). Studies have also demonstrated that under the same circumstances, higher stress is experienced by women with PMS in the luteal phase than in the follicular phase (51). Moreover, the hypothalamus–pituitary gland–adrenal gland axis and the autonomic nervous system are the two main networks systematically associated with stress, and the dopamine (DA) system has been proven to play a key role in the response to stress (52). A study on rats showed that exposure to long-term stress resulted in a substantial reduction in the activity of the DA neuron population activity (53), and disruption of the DA system led to dysfunctional reward processing (54, 55). Second, the response bias in this task is associated with basal ganglia responses and feedback-related negativity toward reward feedback (56) and is reported to be impaired by a single dose of dopamine agonist [pramipexole; (25)]. Thus, the behavioral abnormalities observed in this study may have been induced by the abnormal activity of the DA system. In summary, menstruation can be viewed as a long-term, periodic, negative event for women with moderate-to-severe PMS. The tracts and structures of the DA pathways associated with the reward processing in the luteal phase are altered, thus inducing dysfunctional reward processing, which was measured using a PRT in this study.

Notably, according to the results for reaction time and discrimination (no group difference), the difference in response bias between the two groups was not due to general task deficiencies. According to the formula used for response bias and the results of hit rate tests, a low hit rate for rich stimuli can be considered the main cause of PMS. In other words, women with moderate-to-severe PMS tend to recognize rich stimuli as lean stimuli in the late luteal phase, as do patients with BPD (27) but not patients with MDD (26). To further investigate why women with moderate-to-severe PMS tended to have lower response bias than do women without PMS in the late luteal phase, we analyzed the miss rate probabilities for rich stimuli as function of the preceding trials. The results revealed that in both phases, when the preceding trial involved a rewarded lean stimulus, the women with moderate-to-severe PMS exhibited a trend of having a higher miss rate for rich stimuli than did the women without PMS. These results were similar to those reported in studies on patients with BPD (27). Higher miss rates for rich stimuli were observed when rich stimuli were preceded by rewarded lean stimuli, indicating that women with moderate-to-severe PMS were hindered in their development of response bias toward more frequently rewarded stimuli after receiving rewards for less advantageous responses. Therefore, the depressive tendencies of the PMS group in the late luteal phase may have resulted from their excessive attention to unusual stimuli (rewarded lean stimuli), which impaired their ability to integrate and reinforce information. Furthermore, similar results observed in women with moderate-to-severe PMS and patients with BPD indicated a similarity between these two types of emotional disorders. In other words, moderate-to-severe PMS may not merely entail general depression, but it may be a depressive and manic emotional disorder. Qiao et al. (57) investigated 731 women with PMS in China. Among these women, 519 (70.9%) had irritability symptoms (hypomania-related symptoms) and 234 (32.0%) exhibited melancholia and crying (depression-related symptoms). Given that PMS commonly occurs as a comorbidity in BPD-II, PMS may be a cluster of emotional symptoms rather than symptoms of depression alone. However, the results of the present study were only marginally significant, but they did not reach a significant level. Whether PMS tends more toward depression or BPD warrants further in-depth investigation before conclusions can be drawn.

Furthermore, the altered reward responsiveness and lower hit rate toward more frequently rewarded stimuli in the late luteal phase remained when statistically controlling for the SHAPS scores, indicating that those deficits might represent a stable characteristic in women with moderate-to-severe PMS, similar to MDD (58). However, the results of the probability analyses were nonsignificant after controlling for the SHAPS scores, which indicated that the marginally significant results in the analysis of variance test for the rich stimulus miss rate were driven by anhedonia. Pizzagalli et al. (26) reported that in the MDD sample in their study, neither response bias at Block 3 nor response bias learning (Block 3 – Block 1) were correlated with anhedonic symptoms. However, anhedonic symptoms were positively correlated with rich stimulus miss rates, even after controlling for anxiety symptoms and general distress. In summary, we deduced that blunted reward responsiveness represents a trait-like vulnerability in patients with affective disorders. However, the rich stimulus misses rates were influenced by the participants’ anhedonia.

Notably, some critical findings (particularly the results of probability analyses) only exhibited a statistical trend. The reasons are as follows: first, PMDD, a severe form of PMS that is not PMS itself, is included in the Diagnostic and Statistical Manual of Mental Disorders, Fifth Edition; however, women with PMS do not meet the criteria for mental disorder, therefore, the impairment of functions (such as reward processing) is not as serious as that observed in depression and BPD (26, 27); second, although the present study presents an adequately well-designed paradigm, studies on depression and BPD have also reported marginally significant results (26, 27), therefore, behavioral indicator tests for assessing reward processing are likely to be less sensitive than the direct assessment of reward processing through neurological and brain imaging techniques (e.g., functional magnetic resonance imaging, fMRI). Furthermore, we conducted a separate analysis after a nonsignificant interaction was observed in probability analyses. The separate analysis had been done when the interaction was not significant in the previous studies, both in the behavior researches and fMRI researches. For example, in Bogdan and Pizzagalli (24)’s research, three-way ANOVA with Condition (stress, no-stress), Block (1, 2, 3), and Stress Manipulation (threat-of-shock, negative performance feedback) revealed no significant interaction, and then they analyzed the two stressor manipulations separately and found that the main effect of Condition was significant only for the threat-of-shock manipulation. Furthermore, in an fMRI study conducted by Elman et al. (59), in the anticipation phase, the Group by Spinner interaction failed to produce any significant clusters of activation, and the further separate analyses in healthy participants revealed a significant cluster of activation to the good [a spinner that generated a large gain ($10), a small gain ($2.50), or no gain ($0)] minus bad [a spinner that generated a large loss ($6), a small loss ($1.50), or no loss ($0)] spinner that comprised the right nucleus accumbens, caudate, and putamen, while analyses in participants with posttraumatic stress disorder revealed no significant clusters of greater activation to the good minus bad spinner. However, caution is still advised before drawing conclusions.

The present study has limitations in addition to those mentioned. First, the participants recruited in this study were all female undergraduate or postgraduate students. Future studies should recruit women from multiple age groups, such as adolescent girls and menopausal women, to verify and supplement the findings of this study. Second, crucial demographic information that could influence decision-making tasks (e.g., socioeconomic status) was not collected in the current study. Third, the paradigm used only reward reinforcement. Future studies could compare differences in sensitivity to other reinforcement (such as punishment) between women with and without PMS and assess whether differences are related to PMS severity or negative emotional experiences. Fourth, this study used only behavioral measures, and reported association between reward responsiveness and PMS is not extremely strong. Future studies should integrate functional magnetic resonance imaging to examine whether the impaired ability of women with moderate-to-severe PMS to integrate reward stimuli is related to the brain region responsible for processing reward-related information. The Monetary Incentive Delay Task (60) is the most widely used task to probe the neural substrates involved in the processing of reward and punishment in human volunteers.

## Data Availability Statement

The raw data supporting the conclusions of this article will be made available by the authors, without undue reservation, to any qualified researcher.

## Ethics Statement

The studies involving human participants were reviewed and approved by the body for ethical evaluation of research projects at the Department of Psychology—part of the School for Social and Behavioral Sciences at Nanjing University, China. The authors assert that all procedures contributing to this work comply with the ethical standards of the relevant national and institutional committees on human experimentation and with the Helsinki Declaration of 1975, as revised in 2008. Written informed consent was obtained from all individual participants included in the study.

## Author Contributions

All authors have made a significant contribution to this work. LH and LChe collected the data. LH and RZ analyzed and interpreted the data. LH and LCha wrote the current version of the manuscript.

## Funding

This study was supported by the Key Project of Philosophy and Social Science Research in Colleges and Universities in Jiangsu Province (2015JDXM001), NJU National Demonstration Base for Innovation & Entrepreneurship (SCJD0406), Fundamental Research Funds for the Central Universities (14370303), and Nanjing University Innovation and Creative Program for PhD candidate (CXCY18-06).

## Conflict of Interest

The authors declare that the research was conducted in the absence of any commercial or financial relationships that could be construed as a potential conflict of interest.

## Footnotes

1 Referring to the prior studies (26, 27), in order to explore whether PMS group also showed delayed acquisition of response bias toward the rich stimuli, **Block** was also used as an independent variable.
