# Supplementary material for: Reduced Reward Responsiveness in Women With Moderate - to - Severe Premenstrual Syndrome: Evidence From a Probabilistic Reward Task
Source: Front Psychiatry. 2020 Feb 13;11:28. doi: 10.3389/fpsyt.2020.00028 (PMC7031199; doi:10.3389/fpsyt.2020.00028)
Supplement: Supplementary file 1 [file DataSheet_1.docx]

**Supplements**

**1.1. Demographic variables**

The results revealed no group differences for age, BMI, duration of menstrual flow, or length of menstrual cycle (all *t*(57) < 1.42, *p* > 0.05). The descriptive statistics of demographic variables are listed in **Table 1**.

**Table 1** Demographic variables and PMS scale in two group (M±SD)

|  | PMS group (*n*=28) | Non-PMS group (*n*=31) | *t* |
| --- | --- | --- | --- |
| Age | 21.07 ± 2.18 | 21.87 ± 2.14 | -1.42 |
| BMI | 19.85 ± 1.34 | 20.08 ± 2.01 | -0.52 |
| Duration of menstrual flow (days) | 5.68 ± 1.12 | 5.39 ± 1.05 | 1.02 |
| Length of menstrual cycle (days) | 29.68 ± 2.21 | 29.16 ± 3.14 | 0.72 |

Note: BMI refers to body mass index, which was calculated as the participant’s weight in kilograms divided by the square of her height in meters (kg/m^2^). Duration of menstrual flow here refers to the duration of the menstrual phase in a single menstrual cycle. Length of menstrual cycle here refers to the interval between two consecutive menstrual cycles.

**1.2. Questionnaires and hormones**

No significant effects were observed for PANAS-PA (all *F*(1,57) < 1.24, *p* > 0.27) or BPOMS-VA (all *F*(1,57) < 1.88, *p* > 0.18) or BEES–general arousal (all *F*(1, 57) < 2.53, *p* > 0.12). The ANOVA for BEES–general valence revealed a main effect for Group (*F* (1, 57) = 11.44, *p* = 0.001, *η_p_^2^* = 0.17). The ANOVA for the SHAPS revealed a main effect for Group (*F*(1, 57) = 7.15, *p* = 0.01, *η_p_^2^* = 0.11); the scores of women with moderate-to-severe PMS were higher than those of women without PMS.

The hormone level results revealed significant main effects of Phase for progesterone (*F*(1, 59) = 40.66, *p* < 0.001, *η_p_^2^* = 0.42) and estradiol (*F*(1, 59) = 6.33, *p* = 0.02, *η_p_^2^* = 0.10); however, no other effects were observed for these hormones (progesterone: all *F*(1,59) < 2.26, *p* > 0.14; estradiol: all *F*(1,59) < 0.68, *p* > 0.41). The questionnaires and hormones in two group are shown in **Table 2**.

**Table 2** Questionnaires and hormones in two group (M±SD)

|  | PMS group (*n*=28) | | Non-PMS group (*n*=31) | |
| --- | --- | --- | --- | --- |
|  | late luteal phase | follicular phase | late luteal phase | follicular phase |
| BDI | 15.07 ± 7.30 | 15.07 ± 9.95 | 7.42 ± 6.38 | 7.07 ± 5.81 |
| BAI | 42.29 ± 8.16 | 43.36 ± 11.14 | 33.39 ± 4.61 | 32.97 ± 5.78 |
| PANAS-N | 25.75 ± 6.07 | 24.29 ± 5.54 | 20.87 ± 3.51 | 21.52 ± 4.63 |
| PANAS-P | 23.32 ± 4.88 | 22.68 ± 4.79 | 21.74 ± 4.11 | 22.55 ± 4.85 |
| BPOMS-VA | 6.50±3.23 | 6.50±4.71 | 6.37±4.57 | 8.03±4.18 |
| BEES-general valence | 4.61±1.62 | 4.93±1.68 | 5.68±1.49 | 5.94±1.12 |
| BEES-general arousal | 5.07±1.21 | 4.82±1.54 | 4.94±0.93 | 5.32±1.05 |
| BEES-joy | 4.82±1.61 | 4.54±1.77 | 5.81±1.35 | 5.81±1.62 |
| BEES-anger | 4.54±1.43 | 5.04±1.91 | 3.10±1.14 | 3.19±1.38 |
| BEES-fear | 4. 04 ±1.64 | 4.54 ±2.20 | 3.29 ±1.44 | 2.94 ±1.57 |
| BEES-sadness | 5.00 ±1.76 | 4.96 ±1.88 | 3.87 ±1.65 | 3.90 ±1.89 |
| BEES-calm | 4.46±1.73 | 4.32±1.70 | 5.58±1.26 | 5.55±1.79 |
| BEES-disgust | 4.86±1.46 | 4.57±2.04 | 3.13±1.41 | 3.48±1.77 |
| BEES-surprise | 4.71±1.76 | 4.32±1.59 | 4.03±1.74 | 4.29±1.62 |
| SHAPS | 25.36 ± 5.33 | 25.73 ± 4.52 | 22.00 ± 4.56 | 22.81 ± 5.11 |
| progesterone (pg/mL) | 812.44 ± 585.99 | 368.59 ± 168.99 | 967.75 ± 587.83 | 492.79 ± 306.02 |
| estradiol (pg/mL) | 129.52 ± 101.77 | 101.49 ± 72.64 | 146.36 ± 83.74 | 114.56 ± 71.56 |

**1.3. PRT**

**1.3.1. Response bias**

The ANOVA revealed a significant effect for Block (*F*(2, 114) = 40.95, *p* < 0.001, *η_p_^2^* = 0.42) resulting from significantly higher response bias in Block 2 and 3 than in Block 1 (Bonferroni *p* < 0.05). The main effect for Group (*F*(1, 57) = 6.78, *p* = 0.01, *η_p_^2^* = 0.11) was also significant owing to higher response bias in the women without PMS than in those with PMS (0.19 ± 0.06 vs. 0.14 ± 0.08; *Cohen’s d* = 0.71). Furthermore, although the same ANOVA only revealed a trend of Group × Phase interaction (*F*(1, 57) = 3.47, *p* = 0.07, *η_p_^2^* = 0.06), in the late luteal phase, the women without PMS had a higher response bias than those with PMS (0.20 ± 0.08 vs. 0.11 ± 0.09; *F*(1, 57) = 16.93, *p* < 0.001, *Cohen’s d* = 1.06), whereas in the follicular phase, no difference was observed between the two groups (*F*(1, 59) = 0.01, *p* = 0.87). No other significant effects were found (all *F* < 1.02, *p* > 0.37).

The results of ANCOVA for the response bias in the late luteal phase found the main effect for Group (*F*(1, 56) = 13.04, *p* = 0.001, *η_p_^2^* ^=^ 0.19) was significant owing to higher response bias in the women without PMS than in those with PMS. No other significant effects were found (all *F* < 1.32, *p* > 0.26). For the response bias in the follicular phase, no significant effects were found (all *F* < 3.50, *p* > 0.07).

**1.3.2. Discriminability**

No effects involving Group emerged (all *F* < 0.97, *p* > 0.33).

**1.3.3. Reaction time**

The ANOVA revealed a significant Group × Phase × Block three-way interaction (*F*(1, 57) = 7.32, *p* = 0.01, *η_p_^2^* = 0.11). However, simple effect tests revealed no further significant differences between the two groups for any condition (all *F* < 0.82, *p* > 0.37).

**1.3.4. Hit rate**

One ANOVA revealed a significant main effect for Block (*F*(2, 114) = 5.20, *p* = 0.01, *η_p_^2^* = 0.08) driven by a significantly higher hit rate in Block 3 than in Block 1 (Bonferroni *p* < 0.05). Furthermore, the main effect for Stimulus Type was also significant (*F*(1, 57) = 267.59, *p* < 0.001, *η_p_^2^* = 0.82, rich stimulus > lean stimulus).

Critically, although the Group × Phase × Condition interaction only revealed a trend (*F*(1, 57) = 2.90, *p* = 0.09, *η_p_^2^* = 0.05), women without PMS had a higher hit rate than to the rich stimulus in the late luteal phase did women with moderate-to-severe PMS (0.80 ± 0.06 vs. 0.76 ± 0.08, *F*(1, 57) = 5.25, *p* = 0.03, *Cohen’s d* = 0.57). Moreover, a significant Group × Condition interaction was observed (*F*(1, 57) = 5.14 *p* = 0.03, *η_p_^2^* = 0.08), but no group difference was found in both conditions (all *F* < 2.55, *p* > 0.11). No other significant effects were observed (all *F* < 0.89, *p* > 0.41).

The results of ANCOVA for the hit rate in the late luteal phase found the main effect of Stimulus type was significant (*F*(1, 56) = 9.95, *p* = 0.003, *η_p_^2^* = 0.15) owing to the higher hit rate of rich stimulus than lean stimulus. Furthermore, a significant Group × Stimulus type interaction was observed (*F*(1, 56) = 9.00, *p* = 0.004, *η_p_^2^* = 0.14). The group difference was due to the rich stimulus; specifically, women without PMS had a higher hit rate than did women with moderate-to-severe PMS (0.80 ± 0.06 vs. 0.76 ± 0.08, *F*(1, 57) = 5.25, *p* = 0.03, *Cohen’s d* = 0.57). No other significant effects were found (all *F* < 1.09, *p* > 0.34). For the hit rate in the follicular phase, no significant effects were found (all *F* < 2.75, *p* > 0.07).

**1.3.5. Probability analyses**

The previous analyses indicated that women with moderate-to-severe PMS had significantly lower response bias and a significantly higher miss rate (i.e., lower hit rate) for the rich stimuli. To analyze these findings in more detail, we computed the probability of missing a rich stimulus as a function of the outcome of the preceding trial.

The ANOVA for the rich stimulus miss rate revealed a significant main effect for Preceding trial type (*F*(1, 57) = 19.45, *p* < 0.001, *η_p_^2^* = 0.25). A marginal significant effect was observed for the main effect of Group (*F*(1, 59) = 3.30, *p* = 0.08, *η_p_^2^* = 0.06) due to a higher miss rate in the PMS group than in the non-PMS group (0.24 ± 0.08vs. 0.21 ± 0.06; *Cohen’s d* = 0.42). Although the three-way interaction was not significant, separate analyses indicated that in the late luteal phase, when the rich trial was preceded by a non-rewarded lean trial, the miss rate of the PMS group was higher than that of the non-PMS group (0.20 ± 0.06 vs. 0.15 ± 0.10; *t*(57) = 1.99, *p* = 0.05, *Cohen’s d* = 0.61). Furthermore, in both the late luteal and follicular phases, when the rich trial was preceded by a rewarded lean trial, the miss rate of the PMS group was higher than that of the non-PMS group (late luteal phase: 0.31 ± 0.17 vs. 0.23 ± 0.16, *t*(57) = 1.87, *p* = 0.07, *Cohen’s d* = 0.48; follicular phase: 0.27 ± 0.15 vs. 0.21 ± 0.12, *t*(57) = 1.72, *p* = 0.09, *Cohen’s d* = 0.44). No other significant effects were observed (all *F* < 1.41, *p* > 0.24).

The ANOVA for the lean stimulus miss rate revealed a significant main effect for Preceding trial type (*F*(1, 59) = 19.16, *p* < 0.001, *η_p_^2^* = 0.25). No effects involving Group emerged (all *F* < 1.52, *p* > 0.21).

The results of ANCOVA for all the probability analyses found no significant effects (all *F* < 1.88, *p* > 0.18).
